# American Dental Association

**Published:** 1891-02

**Authors:** 


### Reports of Society Meetings.

### AMERICAN DENTAL ASSOCIATION.—THIRTIETH ANNUAL MEETING, EXCELSIOR SPRINGS, MO., AUGUST 5 TO 8, 1890.

(Continued from page 41.)

### *Third Day's Proceedings.*

THE third day's proceedings of the American Dental Association commenced at the Excelsior Springs, Mo., Opera-House, Thursday, August 7. President M. W. Foster, of Baltimore, Maryland, in the chair.

Dr. Crouse, of Chicago, made a motion that the special order at 7.20 P.M. be the election of officers of the Association.

The motion was carried.

Section 3 was then passed, and Section 4, the Section of Histology and Microscopy was called. Dr. W. X. Sudduth, of Minneapolis, secretary of the section, reported two papers for the evening session, one to be furnished by Dr. Hunt, on the blood-supply of the teeth, the other by Dr. Sudduth, on "Tumors of the Mouth." A brief discussion as to the length of the time that the papers were to be allotted, and as to whether the points presented in the papers were new, was indulged in by Dr. Sudduth, Dr. Truman, Dr. Patrick, Dr. Rhein, Dr. Baldwin, and Dr. Crawford.

Section 4 was passed for the time being and Section 5 called. Dr. Harlan, chairman of Section 5, the Section of Materia Medica and Therapeutics, made the report. The only paper before the section for consideration, and the only one to be considered by the Association, was that upon "Carbolic Acid," by Dr. A. W. Harlan, of Chicago.

In commencing, the speaker gave the history of carbolic acid. It was first discovered by Runge, 1834. He noted the observations of writers upon the subject. The properties of carbolic acid as a disinfectant were then considered; the form that pure phenol assumes; the long, colorless needles. Absolutely pure carbolic acid was first produced in 1871, by Church. He then considered the anæsthetic properties of carbolic acid. It is so powerful and so irritating that it must be used with the greatest care. John Dougall said, as early as 1870, that carbolic acid was not a powerful germicide. The genuineness of the acid can easily be tested by shaking a drachm of the acid with a pint of warm water. The water will dissolve the pure acid, but will retain the dead oil undissolved. The speaker next alluded to the preparation of synthetic carbolic acid. All had been previously prepared from coal-tar. The price of the synthetic acid is quite reasonable. Experiments have been made to test the relative disinfecting power of synthetic carbolic acid, the comparison being made with the ordinary carbolic acid. It was found that there was very little difference between the two acids. One of the advantages of the synthetic acid is its greater solubility. Carbolic acid has been of great value to modern surgery. It has been used by surgeons and by dentists as much or more than any other drug. Of all the remedial agents, carbolic acid and nitrous oxide have been granted the greatest amount of space in the dental and medical journals. Dr. Harlan then gave the following reasons why the use of carbolic acid should be discarded by dentists:

1. Because of its deleterious action on the pulp: oil of cloves or cassia is preferable to carbolic acid.

2. As a root-canal dressing it is, because of its great solubility in water, not to be depended upon.

3. It is not a chemical disinfectant when brought in contact with sulphuretted hydrogen.

4. When it comes in contact with the dentine it is absolutely useless.

5. Unless combined with oils it is valueless.

6. Its beneficial action is only temporary.

7. As an agent for injection it is without value if there are fine roots that hold fragments of pulp.

8. It does not possess embalming properties.

In conclusion, Dr. Harlan said that the fact that so many pulps live when treated with carbolic acid only tends to prove how nature can be abused and yet continue in its wonderful properties. He held that carbolic acid should take its proper place as a spray or as a local anæsthetic.

Section 5 was next passed, and Section 6 was called. Dr. H. A. Smith, of Ohio, chairman of the section, made the report. He said that in 1882 a resolution was adopted at the Cincinnati meeting, and at the last meeting some action had been taken looking to the tabulation of prehistoric crania. The adoption of a system of dental notation seems to be absolutely necessary. He held that this examination of the remains of prehistoric crania would have a great value to dentistry and to science. It would determine whether certain evolutionary theories may or may not be reinforced. He said that Dr. Patrick, of Belleville, Ill., had been selected by the Illinois State Dental Society to take charge of the cranial examinations for that organization. He thought that the American Dental Association ought to be congratulated upon having in its ranks one so able and worthy to carry forward a work of this character.

Dr. Patrick then gave a review of his efforts and researches in this direction. He stated that the report that he had to make to the association was supplemental to what he had already remarked at other meetings. He said that this examination was something that he had contemplated for many years. He was desirous of assistance in this particular field of investigation. In this as in other fields of scientific research and labor one man cannot reap all the honors. He had been working at this project and gathering collections of prehistoric crania for twenty years. He reminded his hearers that what it takes one man twenty years to accomplish can be accomplished by twenty men working one year. He said that it was necessary to adopt a system in this work. The crania should be put up and carefully labelled in cases. The diminution in the size and the number of the teeth in the case of dwarfs should be noted. In the case of giants, the augmentation in the size and the number of the teeth should be carefully observed. The speaker then gave the various heads under which the collections are to be divided. He also gave the various points to be guarded against and examined into. Mutilation and fracture should be recorded. Diagrams will be furnished to all who desire to help in this work by the committee. The symbols can be recorded on the cards, and the committee will add up the totals when they come in. The dental like the medical profession is indebted to the earnest work of men laboring along these lines since the fifteenth century. John Hunter, one of the earlier writers upon the teeth, accomplished his work so well and observed things so closely that nothing that he has done has ever been undone.

When a thorough examination is made of the prehistoric crania of the country the dental profession will be rendering to the anatomist and the physiologist vast assistance. We will be returning something for the benefits that we have received from others. When completed, we will have a cloud of witnesses to testify to the truth of many beliefs and theories. These tables will either approve or disapprove much that is claimed in science. It may clip the wing-feathers of some who desire to fly, but we cannot help that. They must walk the solid earth until content to investigate in a scientific manner. All we ask, all we desire, all we aim at, is the truth, let it hurt whom it will.

Dr. W. S. How, of Philadelphia, secretary of Section 6, next presented a paper on the "Hillischer System of Dental Notation." He said that the *Dental Cosmos*, in 1885, published an article urging the use of symbols in dentistry to describe the teeth. The subject received but little attention in this country at that time, but at the Dental Congress at Paris, last year, "Dental Stenography," as it was called, received favorable consideration. M. Grosheintz communicated a plan for an international system of dental notation. His article attracted considerable attention and was widely commented upon. He gave an account of the various systems proposed in this country and abroad, and described at length the advances made in the art of numbering and classifying the teeth. He congratulated the members of the profession upon the fact that a commencement, at least, had been made in this direction. He thought that the value of these classifications to dentistry and to science would be manifest to all. He demonstrated, in closing, the value of such a system to the different dentists of the country when corresponding with each other regarding different and difficult cases.

Dr. Ottofy, of Illinois, briefly addressed the convention upon implantation. He said that the oldest case that had come under his observation was three years and six months. The tooth was in a perfectly normal and healthy condition until June 1 of this year, when it dropped out. An artificial substitute has since been employed. Dr. Fletcher is having a paper read before the International Dental Congress at the Berlin meeting this year. He has implanted teeth into the hind leg of a goat. The oldest case is over a year old, and the most recent experiments over four months old. In all of these cases absorption has taken place. Photographs have been made showing the implantation to have been made as claimed by Dr. Fletcher.

Dr. Thompson, of Kansas, thought that it did not matter so much what system of notation is adopted, so long as some system is taken up by the dentists and made universal. He hoped that the American Dental Association would take steps looking to the adoption of something in this direction.

Dr. Smith, of Ohio, thought that some one system should be adopted, and the earlier that action is taken upon this subject the better. He was of the opinion that the system adopted by France and Germany could be used with good effect.

Dr. Smith, of Colorado, then proceeded to explain by means of the black-board his system of dental notation. He had adopted with good results the use of horizontal and perpendicular bars. These, placed in different positions, indicated the different teeth and the characteristics of the teeth.

Dr. Ottofy, of Illinois, introduced a resolution, which was adopted, requesting the committee having in charge the Dental Congress to be held at Chicago in 1893 to ask the dentists of the world to submit schemes of dental notation, to the end that some one system may be selected for general use after that date.

Dr. Ottofy further suggested that Dr. Patrick should be given all possible aid in the examination of prehistoric crania by the members of the society. Dr. Noble volunteered to take charge of the work at the national capital, and promised to make thorough researches in the museums at Washington.

Section 6 was then passed, and Section 7, the Section of Anatomy, Pathology, and Surgery was next called. Owing to the absence of the chairman, the report was presented by Dr. Rhein, of New York, the secretary. In presenting his report he made an appeal to the members of the profession in convention assembled to encourage, by every effort in their power, the study of oral anatomy in the dental colleges. He thought it hardly creditable that the students of to-day have so slight a knowledge of oral surgery, and he urged that an increased attention should be paid to it in the educational institutions devoted to the teaching of dentistry.

Two papers referred to Section 7 were next presented by Dr. Rhein. The first was by Dr. A. C. Hugenschmidt, of Paris, France, on the "Occasional Origin of True Chronic Alveolar Abscess in Teeth with Living Pulps." In this paper Dr. Hugenschmidt related a history of three cases in which there was a distinct alveolar abscess in two cases, opening outside at the end of the roots, and where he had to destroy the vitality of the pulps by arsenic, and in one case an injection of cocaine before he could extirpate the pulp. He attributes the etiology of the cases to the presence of some trouble which would ordinarily cause pulpitis and in most teeth be followed by strangulation of the pulp at the apex of the root. But in these cases, on account of the large openings at the apices of the roots, the inflammatory proceeding goes on without necessarily causing death of the pulp. One of the features of these cases was the lack of pain, which ordinarily accompanies true alveolar abscess. The third case was where, in a superior molar in which the pulp had been extirpated from the palatal and anterior buccal root, pus was found at the extremity of the posterior root, although the pulp was found in a living condition.

Dr. M. L. Rhein read a paper upon the amputation of roots as a radical cure in chronic alveolar abscess: in pyorrhœa alveolaris complicated by alveolar abscess. Among other things he said,—

"The removal of a portion or the whole of the root of a tooth has long been advocated by a few men of our profession as a means of restoration to health of that class of teeth which do not yield to milder medical treatment.

"Like many other operations in oral surgery which naturally come within our sphere of practice, it seems to have received little or no attention from those who have made our literature, and text-books are singularly silent concerning this important method of procedure.

"In the majority of cases the operation is one presenting little difficulty. There are no dangerous anatomical points to be avoided except when working under the antrum of Highmore or in the region of the mental foramen.

"The instruments required are a sharp, spear-shaped drill, with which a hole is drilled through the process passing through the root on a line with the canal. This is followed by a new fissure-drill, but which, worked laterally in both directions, readily severs the end of the root.

"Usually no anæsthetic is required. We must, however, occasionally resort to one where the operation is of considerable length and very painful. Under such circumstances a physician should be summoned who will administer an anæsthetic, leaving the dentist undisturbed in his surgical interference.

"It is well known that the longer an alveolar abscess exists the greater the damage to the surrounding tissue. The injury that the general system receives from the absorption of a certain amount of pus must not be ignored.

"About the apices of the root is found an ever-enlarging zone of diseased tissue. This can well be called the apical space, and acts as the hot-bed for the formation of pus, ichorous or not, as the case may be. The accustomed effect of this is to denude the apex of all circulatory supply, and in time to bring about a necrotic condition of the root which gradually extends towards the crown of the tooth until, eventually, the entire root becomes necrosed. Treatment through the canal of the root for the cure of this disease is useless after necrosis has once attacked the apex. The operation of entering through the gum and burring the diseased tissue is uncertain in its results. Having filled the root or roots, the diseased portion is excised. This is at once followed by the vigorous use of the bur in the surrounding pathological tissue, and an immediate and radical cure is the result.

"All that is requisite is to see that the operation has been performed under true aseptic conditions and that the parts are kept so until the wound has entirely healed.

"One of the worst conditions that we meet with in the various aspects of pyorrhœa alveolaris is where, through the ravages of the disease, death of the pulp has ensued and there is added to the original septic matter the pus from the broken-down pulp. This condition generally takes place without any warning to the patient; in fact, it is impossible to learn at what time the death of the pulp takes place.

"The powerful nature of the double septic condition soon causes absorption of the end of the root to progress more or less rapidly, so that the purulent matter finds a free escape from the root and passes out through the channel furnished by the pyorrhœa pocket.

"Consequently the diagnosis of such a pulpless tooth becomes a very difficult matter. The color remains good; even the electric mouth-lamp generally fails to indicate that the pulp is dead, because the canal is filled with purulent matter of such a watery consistency that it is rendered as translucent as though traversed by a living pulp. The only reliable diagnostic sign is to isolate the tooth by means of the rubber dam, and applying intense cold or heat. The smallest amount of the spray of chloride of methyl is admirably adapted for this purpose. We have all experienced the hopelessness of treatment of such teeth, and a course of procedure which will enable us to preserve them for useful service may perchance be welcomed by the profession."

Dr. Rhein cited five cases, admirably arranged, to bring out the points presented in the paper. Two of the most interesting cases are here presented.

Mr. H., aged about thirty, presented himself in 1882 to have his mouth put in good order. A very long and firmly-embedded root was all that remained of the right superior cuspid. As far as could be ascertained the crown had been broken off for some years and at intervals the root had given rise to considerable pain. There was no evidence of any abscess.

Having thoroughly cleansed the canal, the apex was found to be open and the root slightly absorbed around the edges. A forty-per-cent. solution of chloride of zinc was pumped through the apex, which was then sealed with chlora-percha one-third of the length of the root, and the remaining portion was filled with oxy-phosphate of zinc.

Ten days later the patient returned suffering severely from all of the symptoms of alveolar abscess except the swelling. The external parts appeared about normal, and it seemed impossible to bring about resolution. The temperature of the patient was now above 100° F., and a diagnosis was made of the blind abscess with evidences of slight septicæmia. The patient was at once put on a tonic and antipyretic treatment, and it was determined to amputate the end of the root.

Chloroform having been administered, an incision was made in the gum over the apex of the root, and a piece of tissue about three-eighths of an inch was removed. A spear-shaped drill was then passed through the process, and with a new fissure-bur one-eighth of an inch of the apex was severed from the rest of the root. It was with great difficulty that this was removed, and not until a considerable portion of the alveolus had been drilled away; attached to the root was a sac about half an inch long. The entire space was then thoroughly burred. It was washed out with a carbolized solution and allowed to heal under iodoform dressing. Had we had our present knowledge of the various germicides, carbolic acid would have been discarded for something more efficacious. A crown was subsequently attached to the root and is doing good service to-day.

Mr. T., aged about fifty-three, of vigorous and healthy constitution. He had always taken fair care of his teeth, none of which had been lost. The deposit of salivary calculus had always been dense and rapid in its formation, necessitating very frequent attention to the removal of the same. Having somewhat neglected his mouth, he presented himself in 1889 for treatment. There was an extensive deposit of salivary calculus, on removing which it became evident that the former work of removing the same had been very superficial. There was considerable tumefaction of the gum and a discharge of pus. Local treatment restored his mouth very speedily to a condition of health with the exception of the superior left first molar.

The palatine root of this tooth was entirely denuded of any covering on its palatal aspect down to the very point of the apex, its nerve-connection having long since been severed. No filling had ever been inserted in the tooth and there was no sign of caries. The rubber dam having been adjusted, a hole was drilled through the centre of the crown and the pulp, which was found to be in a putrescent state, thoroughly removed from the three roots. They were then cleansed and filled as previously described, and a permanent filling inserted over the oxyphosphate to close the opening in the crown.

A fine fissure-bur was then passed through the palatine root close to the crown, and the entire root removed. At the point of amputation another permanent filling was inserted.

Two weeks later the gum on the palatal side was in a healthy condition, all traces of the imprint of the root had disappeared, and the tooth, which had been very loose, was now firm in its socket, supported by the two buccal roots.

This is the most common class of teeth that we meet in which the amputation of the roots is indicated. Not only do we restore to usefulness a loose tooth, but we stop the absorption of a certain amount of purulent matter.

To briefly summarize: Wherever death of any portion of a root has taken place, the simplest cure is to amputate the necrosed portion of the root and the tissue will close firmly about the remaining healthy portion.

(To be continued.)

